# The functional foetal brain: A systematic preview of methodological factors in reporting foetal visual and auditory capacity

**DOI:** 10.1016/j.dcn.2015.04.002

**Published:** 2015-04-15

**Authors:** Kirsty Dunn, Nadja Reissland, Vincent M. Reid

**Affiliations:** a Department of Psychology, Lancaster University, UK; b Department of Psychology, Durham University, UK

**Keywords:** Foetus, Visual perception, Auditory perception, fMRI, fMEG

## Abstract

Due to technological advancements in functional brain imaging, foetal brain responses to visual and auditory stimuli is a growing area of research despite being relatively small with much variation between research laboratories. A number of inconsistencies between studies are, nonetheless, present in the literature. This article aims to explore the potential contribution of methodological factors to variation in reports of foetal neural responses to external stimuli. Some of the variation in reports can be explained by methodological differences in aspects of study design, such as brightness and wavelength of light source. In contrast to visual foetal processing, auditory foetal processing has been more frequently investigated and findings are more consistent between different studies. This is an early preview of an emerging field with many articles reporting small sample sizes with techniques that are yet to be replicated. We suggest areas for improvement for the field as a whole, such as the standardisation of stimulus delivery and a more detailed reporting of methods and results. This will improve our understanding of foetal functional response to light and sound. We suggest that enhanced technology will allow for a more reliable description of the developmental trajectory of foetal processing of light stimuli.

## Highlights

• Differences in parameters of functional foetal brain imaging studies are explored.
• These variations potentially contribute to variation in published findings.
• Where parameter information is provided, there are variations in techniques, and measures.
• Critical aspects of design likely impact response rates and latencies reported.
• For comparisons of data, more aspects of stimulus presentation should be detailed.

## 1 Introduction

This paper explores the relationships between parameters of functional foetal brain imaging studies and how these potentially contribute to variation in published findings. Such a report could lead to important consequences for our understanding of cognitive development before birth. This review focuses on the use of foetal magnetic resonance imaging (fMRI) and foetal magnetoencephalography (fMEG) in recording foetal cortical activation in response to both visual and auditory stimuli. Many of the studies reviewed here are within the literature from a feasibility perspective, containing small sample sizes and techniques that have yet to be replicated. It is therefore highlighted that this is a tentative review of an emerging field.

There is much variability in the amount of description present in methods sections, with some providing detailed information (e.g., Zappasodi et al., 2001) whereas others do not include information such as attrition rate, number of trials present in final dataset or information about the stimuli (e.g., Eswaran et al., 2000). For those that do provide methodological information, there are variations in techniques, methods, and measures. It is possible that this is due to the focus of the field thus far being on the feasibility for delivering stimuli and recording a neural response in a foetal population. Consequently little has been done to address what could be causing variance in results between studies. For example, experimenters have instead focussed on different processing methods for reducing noise, which will contribute to variance (Samonas et al., 1997, Taulu et al., 2004, Vrba et al., 2004). Despite successes in processing methods, disparities in response rates and response latencies between studies remain in the literature. Many other factors can cause variance, which cannot be controlled in a typical within-subjects design, such as distance from stimuli and foetal state (Kiefer-Schmidt et al., 2013). Due to there being so much variation in factors that are difficult to control, such as foetal state, it is even more imperative that we understand the potential variance that is present due to paradigm construction and stimuli.

Despite these forms of variation, it is clear that it is feasible to both present stimuli and record foetal neural responses. Though the body of literature reporting the investigation of foetal response to external auditory and visual stimuli is small, it is now of a size where we can begin to make comparisons with the potential to answer questions on how and why researchers might, or might not, find statistically significant results. It is essential for the field to begin to provide some consistency across studies in terms of methodological factors, such as the stimulus duration, and to report such information in methods sections. Given the large number of uncontrollable variables in foetal research when compared to postnatal work, it seems particularly important to standardise and report as many of the controllable methodological variables as possible. This will enable better comparisons between results of studies as well as potentially reducing the variance in reports of foetal neural response to external stimuli. Further, establishing a consistent, effective methodology could help improve attrition rates leading to more efficient data collection. This is particularly important for a field with such an inherently difficult target sample.

## 2 Methods

### 2.1 Literature review and eligibility criteria

A literature search was performed in October 2014 using Web of Science with a topic search of “fetal” and “fMRI”, “fetal” and “fMEG”, Visual Evoked Response (VER) and Auditory Evoked Response (AER).

Studies were included if they were investigating the foetus utilising fMRI or MEG methodologies, reported between 1985 and 2014 in an English language journal. No limitations were used.

**Fig. 1:**
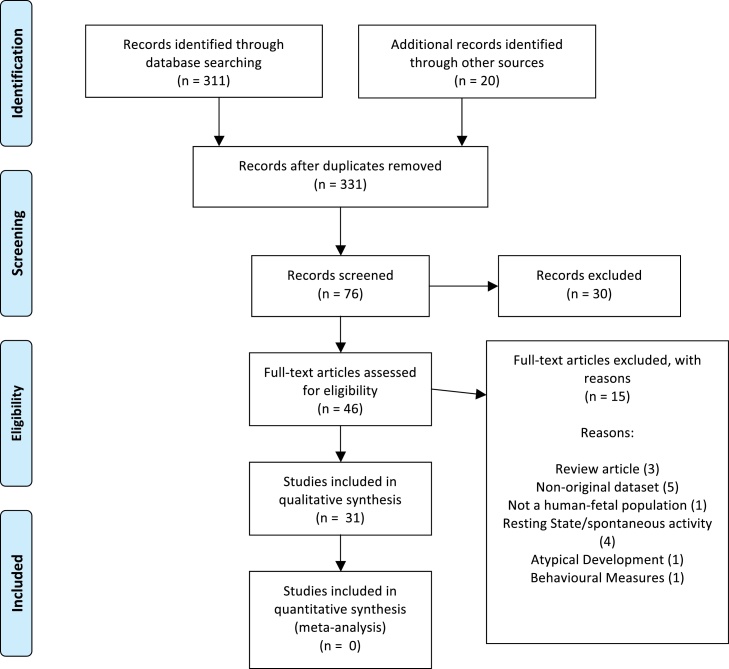
The study selection process as recommended in the PRISMA guidelines.

In selecting studies for inclusion, the PRISMA (2009) process for systematic review was followed as outlined in Fig. 1. Abstracts of articles were screened and those highlighting a response to external visual and/or auditory stimuli were reviewed in full if the results were obtained from singleton pregnancies with no complications. Additionally, potentially relevant journal articles were sought by searching citation lists of the articles that met the inclusion criteria. Papers were reviewed and assessed for exclusion under the following criteria: (a) review articles, (b) purely comparison of data analysis techniques, (c) non-foetal sample, (d) sample assessing atypical development, and (e) non-visual or auditory stimuli.

### 2.2 Data extraction

Dependent measures related to response statistics and these were comprised of the final number of participants and/or recordings included in the final sample, the rate of response (%), and the number of trials included in the analysis post artefact removal. Statistics were extracted manually. For studies using MEG methodology, latency data were extracted. Response rate refers to the percentage of the foetal sample that provided a response (in terms of the number of participants or number of trials included in their analysis). We have classified this in terms of poor (<30%), moderate (30–65%) and good (>65%) response rate relative to the spread of response rates across the studies. Response number refers to the number of participants or recordings entered into analysis post artefact rejection. Response latency for fMEG refers to the time it takes the foetus to respond to a stimulus and has been classified as fast (<150 ms), moderate (150–350 ms) and slow (>350 ms). This final classification is most relevant for articles that utilise an oddball paradigm.

Data was calculated where possible for missing values. Foetal response and latency statistics were compared on the basis of a number of data items including, modality of stimuli, cognitive ability addressed, methodology (fMRI or fMEG), stimuli delivery method, stimulus duration, inter-stimulus interval (ISI), ratio of stimulus duration to ISI, sample size (recruited and final), gestational age (GA), form of stimuli, number of presentations, total time of the study, frequency/wavelength, and volume/intensity.

In addition to the above measures, selection bias within the field may affect the results and conclusions of this review. Statistically significant results are more likely to be reported than non-significant results (Sterne and Egger, 2001). Further, studies with smaller samples are less likely to yield significant results unless results are strong. This is particularly relevant for this review as many of the studies report small sample sizes.

## 3 Results

Fig. 1 details the study selection process as recommended in the PRISMA guidelines. Characteristics of all studies discussed are presented in Table 1, Table 2, Table 3. Characteristics of studies that are not discussed but were nevertheless analysed are presented in Table 4, Table 5, Table 6. Tables are not provided for visual fMRI studies as just one of this kind met the eligibility criteria. In this review a number of studies meeting the general inclusion criteria had to be excluded on the following grounds. Any of the first feasibility studies investigating fMEG response to auditory stimuli (Blum et al., 1985, Wakai et al., 1996, Eswaran et al., 2000) were excluded as Lengle et al. (2001) state environmental noise may have confounded the reported results due to the methods of sound delivery. Early studies also used very few channels due to limitations of the technology. In comparison, other fMEG studies used up to 151 channels (Eswaran et al., 2005, Draganova et al., 2005). McCubbin et al. (2007) were also excluded on the basis of reporting a substantially larger variance in response latency than other studies, which could suggest a different level of acceptance of noise within the obtained fMEG data.

**Table 1:** Variables discussed regarding visual MEG studies. Bold italicised items have been calculated by the current authors.

Visual MEG											
		Measures						Variables			
Authors	Date	Number of trials included	Response rate	Average latency (ms)	Early response	Number of late responses	Significant age effect?	Brightness	Number of presentations	Duration (min)	Style of presentation
Eswaran et al.	2002b	Not stated	***40%***	180–390	No	No	Yes	8800 lx	180	6	Flash
Eswaran et al.	2004	Not stated	28–32 (60%) 32–36 (70%) 36–40 (28%) (b)	28–32 (248.6) 32–36 (196.2) 36–40 (115). (b)	***No***	***2***	Yes	8800 lx	180	6	Flash
Eswaran et al.	2005(2)	Not stated	68%	254.7	***No***	***No***	No	8800 lx	180	6	Flash
McCubbin et al.	2007	Not stated	***92.59%***	180–770	No	Yes (a)	Inconclusive	***35*** ***mW (approx. 4000*** ***lx)***	150	8	Flash
Sheridan et al.	2008	Mean = 30	29.00%	350	***No***	***4***	Not addressed	***35*** ***mW (approx. 4000*** ***lx).***	360	30	Flash set
Matuz et al.	2012	Min = 70	Long ITI (45.4%); short ITI (23.8%)	Long ISI (414); short ISI (330); tone significantly shorter than flash	***No***	***10 (light), 2 (sound)***	More bimodal responses in older foetuses	***35*** ***mW (approx. 4000*** ***lx) followed by 110*** ***dB***	450	20–30	Flash set and pure tone

(a) Statistic cannot be calculated.

(b) Ages represent gestational age of foetus in weeks.

**Table 2:** Variables discussed regarding auditory MEG studies. Bold italicised items have been calculated by the current authors.

Auditory MEG											
		Measures					Variables				
Author	Date	Response rate	Response latency (ms)	Early	Late	Age effect (c)	Paradigm chosen	Delivery method	ISI: stimulus duration (d)	Number of stimuli	Duration (min)
Lengle et al.	2001	54%	100, 210 and 350	No	No	Negative correlation	Simple	Tube	***1:25***	600	***13***
Zappasodi et al.	2001	***50%***	200–300.	No	No	Not addressed	Simple	Tube	***1:5***	230	***13***
Schneider et al.	2001	***51.56%***	56–294 (4 components)	***Yes (a)***	***No***	Not addressed	Simple	Tube	***800 (1:16)*** ***1000(1:20)*** ***1200 (1:24)***	500	***8–9***
Schleussner et al.	2001	56%	30 (300) 40 (157)	***15***	***4***	Negative correlation	Simple	Tube	***800 (1:16) 1000(1:20)*** ***1200 (1:24)***	500	***8–9***
Eswaran et al.	2002a	Sample (80%) recordings (50%)	***260.25***	***No***	***No***	Not addressed	Discrimination (b)	Tube/bag	***1:10***	350	***6***
Schleussner et al.	2004	(a)	56–394 (5 components)	***10***	***No***	Negative correlation	Simple	Tube	***1000 (1:20)*** ***800 (1:16)*** ***1200 (1:24)***	500	***8–9***
Schleussner and Schneider	2004	77%	Range (56–394 ms) Mean (210–394).	***13***	***No***	Negative correlation	Simple	Tube	***1:20***	500	***8–9***
Huotilainen et al.	2004	***Standard (41.18%); Deviant (70.59%)***	Standard (230) MMN (332)	***No***	***5***	Not addressed	Discrimination	Tube/funnel	***1:8***	Not stated	(a)
Draganova et al.	2005	60%.	Standard (260) MMN (321) LDN (458)	***No***	***10***	Not addressed	Discrimination	Tube/bag	***800 (1:8)*** ***500 (1:5)*** ***1100 (1:1100)***	***Approx. 700***	***10.***
Eswaran et al.	2005(1)	62%	264.6.	***No***	***No***	Not addressed		Tube/bag	***1:2***	350	6
Holst et al.	2005	83%	27–31 (288) 32–35(251) 36–39 (197)	***No***	***3***	Negative correlation	Discrimination (b).	Tube/balloon	***1:4***	144	***6***
Draganova et al.	2007	STD (56%) MMN (46%) Early (32%) LDN (32%)	224 (STD) 427 (MMN)	***No***	31	No	Discrimination	Tube/bag	***1:1*** ***1:8***	***Approx. 600***	12
Sheridan et al.	2010	75–90%	Long ISI (360.66 and 641.07) Short ISI (366.8)	***No***	***17***	Trend	Discrimination	Tube/bag	***1:1*** ***1:4***	Short (721) long (735)	11/14
Muenssinger et al.	(2013)	Sample 1(56%) Sample 2 (80%)	Not addressed	Not addressed	Not addressed	Not addressed	Discrimination	Tube/balloon	***1:4; 1:5*** ***1:2; 1:5***	700	12–17
Schleger et al.	2014	***73.91%***	Early (207) Late (427)	(a)	Yes (a)	Negative correlation	Discrimination (number)	Tube/bag	***1:2***	Not stated	(a)

(a) Number cannot be calculated.

(b) Discrimination design in attempt to avoid habituation but discussed only response to standard stimuli discussed.

(c) Negative correlation with age occurs when latency reduces throughout gestational age.

(d) Calculated from the inter-stimulus duration to stimulus duration ratio.

**Table 3:** Variables discussed regarding auditory fMRI studies. Bold italicised items have been calculated by the current authors.

Auditory fMRI						
		Measures	Variables			
Author	Date	Response rate	Sample size	Age range (weeks)	Stimulus type	Intensity (dB)
Hykin et al.	1999	***66.60%***	3	38–39	Maternal speech (nursery rhyme)	100
Moore et al.	2001	***71.42%***	7	37–41	Spanish guitar music	85
Fulford et al.	2004	***46.66%***	15	36 onwards	Vibroacoustic stimulation	95–100
Jardri et al.	2008	***66%***	3	28–34	Pure tone	100
Jardri et al.	2012	***100%***	3	33–34	Voice	100

**Table 4:** Additional variables analysed from auditory MEG studies.

Auditory MEG									
Author	Date	Recruited sample	Final sample size	GA (weeks)	Stimulus duration (ms)	ISI (ms)	Frequency (kHz)	Intensity (dB)	Number of trials included in analysis
Lengle et al.	2001	19	Not stated	29–40	50	1250	1.5	100	Not stated
Zappasodi et al.	2001	12	12	36–40	500	2631	0.5 1.	103	200
Schneider et al.	2001	27	21	29–41	50	Sample 1 (800–1200) Sample 2 (1000)	0.5	100	Not stated
Schleussner et al.	2001	27	Not stated	29–40	50	Sample 1 (1000) Sample 2 (800–1200)	0.5	100	Not stated
Eswaran et al.	2002a	10	T1 – 10 T2 – 5	T1 – 30–35 T2 – 36–40	100	1000	0.5 1(20%)	120	Not stated
Schleussner et al.	2004	Not stated	38	***27–40***	50	Sample 1 (1000) Sample 2 (800–1200)	0.5	100	Not stated
Schleussner and Schneider	2004	38	12	27–40	50	1000	0.5	100	Not stated
Huotilainen et al.	2004	17	17	35–40	100	800	0.5 0.75	85–90	>150
Draganova et al.	2005	12	12	33–36	100	Sample 1 (500–1100) Sample 2 (800)	0.5 0.75 (12%)	110	STD (300) Deviant (40)
Eswaran et al.	2005(1)	11	11	28 onwards	1000	2000	0.5 1 (20%)	120	Signal to noise ratio at least 2:1
Holst et al.	2005	18	16	27 onwards	500	2000	0.5 0.7/1 (20%)	120	Not stated
Draganova et al.	2007	18	18	28–39	100	700–900	0.5	120	STD (600) Deviant (70)
Sheridan et al.	2010	22	20	29–38	70	70 300	0.5. 0.5 (15%)	120	Not stated
Muenssinger et al.	(2013)	Sample 1 (25) Sample 2 (12)	Sample 1 (14) Sample 2 (5–10)	Sample 1 (35) Sample 2 (25)	Tone (500) White noise (1000)	2000–2500	0.5. 0.75	95	Not stated
Schleger et al.	2014	30	23	30–39	70	150 ITI (1000)	0.5	95	Not stated

**Table 5:** Additional variables analysed from auditory fMRI studies.

Author	Date	Number of presentations	Total time (mins)	Delivery method	Stimulus duration (ms)	Ratio of stimulus duration:ISI (a)	ISI (ms)	Frequency (kHz)	Response number	Number of trials included in analysis
Hykin et al.	1999	18	***9***	Speaker.	15,000	***1:1***	15,000	N/A	2	Not stated
Moore et al.	2001	30	***15***	Headphones	15,000	***1:1***	15,000	N/A	5	Not stated
Fulford et al.	2004	30	***10***	Acoustic stimulator	4000	***1:4***	1600	N/A	7	Not stated
Jardri et al.	2008	12	***7***	Headphones	21,000	***4:7***	12,000	0.5 0.7 0.9	2	Not stated
Jardri et al.	2012	9	***7***	Sound belt	21,000	***4:7***	12,000	0.5 0.7 0.9	3	Not stated

(a) Calculated from the inter-stimulus duration to stimulus duration ratio.

**Table 6:** Additional variables analysed from visual MEG studies.

Authors	Date	Recruited sample	Final sample size	GA (weeks)	Stimulus duration (ms)	Ratio of stimulus duration: ISI (a)	ISI (ms)	Wavelength (nm)
Eswaran et al.	2002b	17	10	28–36	33	***1:60***	2000	625
Eswaran et al.	2004	12	9	28 onwards	500	***1:3***	1500	625
Eswaran et al.	2005(2)	11	11	28 onwards	33	***1:60***	2000	625
McCubbin et al.	2007	27	25	30–36	500	***1:7***	3500	630
Sheridan et al.	2008	25	25	29–37	500	***2:5***	200. ITI (10,000)	630
Matuz et al.	2012	Long (26) Short (14)	Long (23) Short (14)	30–38	500	***1:4***	2000 Long ITI (12,500) Short ITI (4500)	630 nm, followed by 500 Hz

(a) Calculated from the inter-stimulus duration to stimulus duration ratio.

Not all measures are highly variable with some measures proving to be relatively stable across research reports. Problems arise when comparing studies due to missing data. A number of articles failed to report methodological details and/or specific results. For thorough analysis, we endeavoured whenever possible to calculate missing data. This was not always possible, however, and gaps still remain leading to inconsistencies in the level of analysis that is possible. Additionally, with the exception of one study, most fMRI studies used a small sample size (*M* = 7) and a relatively restricted gestational age (GA, *M* = 4 weeks). In contrast, the majority of the fMEG studies used larger small sample sizes (*M* = 13) and had a larger gestational age range (*M* = 7 weeks). This causes fundamental forcomparisons between methodologies, with the effect that such comparisons are not meaningful.

### 3.1.1 Visual processing-MEG

The brightness of the stimuli presented to the foetus is a particularly crucial factor when assessing foetal visual response. This is due to the scattering and absorption properties of the matter between the light source and the foetal eye, including the maternal skin, adipose and muscle tissue, and uterus (Bearden et al., 2001). In a model of light penetration to the uterus, Del Guidice (2010) predicted that 0.1–1% of the external illuminance should reach the foetus. In our comparisons of studies using fMEG, a clear difference in the percentage of foetal response was found between those studies that used a 35 mW (Sheridan et al., 2008, Matuz et al., 2012) compared with a much better response rate for those who reported the use of 8800 lux (Eswaran et al., 2002a, Eswaran et al., 2004, Eswaran et al., 2005). Matuz et al. (2012) concluded their poor response rate was the result of the use of a light source that was not bright enough (at 35 mW) to be perceived by the foetus. They recommended a brighter light source for future studies. Converting values in mW to lux for comparison is difficult as they are not simply measurements of the same unit. The former is a measurement of irradiance while the latter is a measurement of illuminance. This makes comparisons of the brightness between the studies problematic. Our calculations showed the former to be approximately half the brightness of the latter, at around 4000 lux based on an average efficacy of 0.25 (35 mW/15 × 10^−4^ m^2^ = 23,300 mW/m^2^ or approximately 4000 lux). It is likely then, that the brightness of the light source can have an impact on foetal response rate to the stimuli and a brightness of 8800 lux is a more appropriate strength to reach the foetus through the maternal tissue. Further, those classified as having a poor response rate delivered light to the foetus using a 3 cm × 5 cm woven panel. In contrast, those classified as having a good response rate delivered light through a much smaller light guide. Therefore, the method of light delivery leading to a smaller surface area of illumination and larger intensity of light may well also explain the improved foetal response rate. Researchers should agree on the same measurement for reporting stimuli brightness as well as delivery methods in future papers in order to establish the most effective methodology for analysis of foetal response to light.

Those that reported use of 35 mW stimuli (Sheridan et al., 2008, Matuz et al., 2012) also reported a longer latency for response at 350 ms (Eswaran et al., 2002a, Eswaran et al., 2004, Eswaran et al., 2005) than those who utilised a light source of 8800 lux (around 250 ms). Additionally, 360–450 presentations of light flashes were presented in studies that used 35 mW of light compared with 180 flashes in those that used 8800 lux. The former of the two kinds of studies were also much longer (20–30 min) when compared to the latter (6 min). Those that used 35 mW were classified as having a poor response rate utilised paradigms designed to assess response decrement rather than the simple presentation of flashes. The issue therefore becomes whether this reduction in response rate reflects a reduction in response via habituation, leading to an overall lower percentage response rate or whether this reflects a reduced, slower reaction to dimmer light presented in the 35 mW studies.

It is also possible that differences in response rate were affected by the level of remaining data following the removal of artefacts caused by movement and noise, such as maternal and foetal heart rate. Low response rates in studies using 35 mW could be the result of a smaller pool of artefact free data compared with those who use 8800 lux.

In addition, studies did not consistently report the number of trials entered into analysis. Comparisons therefore cannot be made between the mW/lux studies. Sheridan et al. (2008) reported a low response rate (29%) despite a high rate of remaining data post artefact removal (79%) indicating an average of 30 included trials in the analysis. Matuz et al. (2012), however, reported entering a minimum of 70 trials in the analysis. Number of trials is therefore unlikely to explain the variation between different study types. Further comparisons between studies cannot be made as the remaining studies did not report statistics of data post removal of artefacts. Therefore the consistency of this amongst other studies cannot be determined. We recommend that the reporting of details related to data pre and post-artefact removal takes place, as is the case in infant EEG studies (e.g., Hoehl and Wahl, 2012), in order that thorough comparisons can be made.

In addition to artefacts noted within articles, we suggest that researchers provide information about foetal eye movement in their analyses. Such an assessment could determine whether the eye is open (Reissland et al., 2015), examine whether the light is aversive and ensure movement is not masking response levels. Researchers have often used ultrasound technology to detect the position of the eye before light delivery (e.g., Matuz et al., 2012, Schleger et al., 2014). Due to recent developments in 4D ultrasound technology, it is now possible to detect not only eye but also eyelid movement before and after stimuli delivery (Reissland et al., 2015) and fMEG measurements.

In MEG research, response rate is often higher in those who tend to report either a large range of latencies or faster response latency than those who report lower response rates. Studies using a repetitive flash design, investigating simple reaction to light, tended to find a short latency of response (Eswaran et al., 2002b, Eswaran et al., 2004, Eswaran et al., 2005). In contrast, those studies that used a set of flashes assessing potential discriminatory processes tended to find a longer latency (Sheridan et al., 2008, Matuz et al., 2012). Based on results of infant research, this could be interpreted as differential processes between simple visual detection and downstream visual processing, such as visual discrimination. This could also be interpreted as the foetus attempting to process a set of visual flashes as a whole compared with continual flashes leading to differences in time taken to process the stimuli. On the basis of the current literature, it would be important to differentiate results as a consequence of presentation style as this factor could activate differential processes for the foetus.

The presentation style of flashes (continual and sets) coincides with a pattern in the number of presentations and length of time of the study. This could lead to a more simple interpretation where those presenting a continual array of flashes also reported fewer presentations (150–180) and a much shorter total presentation time (6–8 min) than those who presented sets of flashes for discrimination (360–450 presentations and 20–30 min, respectively). The majority of studies were consistent in the use of 500 ms as the duration of each stimulus presentation, thereby failing to explain differences in response latency between studies. Based on infant EEG, it may be the case that longer paradigms, with a larger number of presentations, lead to the longer latencies. Infants become neurally habituated during the course of most visual Event-related potential studies (Stets and Reid, 2011). Longer studies compared with shorter studies report “lower” response rates, which also points towards foetal habituation over trials. One interpretation of the current literature is that it is possible that more sophisticated processes in the foetal brain are activated when processing a more complex visual task requiring discrimination. The difference in latency reflects this difference in processing work required of the foetal brain. Because of variations in methodology, including brightness of stimuli, wavelength, and presentation style, it is not possible to assess which factors are primarily responsible for differences in results between studies.

### 3.1.2 Visual processing–fMRI

One study using fMRI has investigated the effect of light on foetal response, which could potentially explain which of these factors, namely brightness, wavelength, or paradigm, caused a difference in response rate (Fulford et al., 2003). Investigating simple responses to light, the authors found a 62% response rate was localised to the frontal cortex in foetuses of at least 36 weeks GA. Since in this study a constant intensity of light was projected, this study does not clarify whether type of presentation resulted in differences. Interestingly, the light (with 1100–1200 lux projected) was much weaker than any of the fMEG studies over a wider illuminated area of 20 cm^2^. Unless fMRI is a more sensitive measure of foetal response than MEG, this contradicts the view that a light source of approximately 4000 lux would provide light that is too weak for a strong response rate to the stimuli. This highlights the critical role of communication between researchers and the consistent reporting of details of stimuli characteristics in aiding future research.

### 3.2 Visual processing: summary

There are relatively few published reports assessing visual functional processing in the human foetus. These studies, however, (Eswaran et al., 2002b, Eswaran et al., 2004, Eswaran et al., 2005, McCubbin et al., 2007, Sheridan et al., 2008, Matuz et al., 2012, Fulford et al., 2003) use similar methodologies in some aspects of their design, such as stimulus duration. It is likely that critical aspects of design, such as the brightness of the light source, area of illumination and the number of presentations to the foetus do have an impact on the response rates and latencies that have been found. Since Eswaran et al. (2004) is the only paper to report differences in these measures across ages and none of the papers reports differences in these measures within a foetus across the time span of the study, it is difficult to conclude which of these factors has an impact on foetal response. More consistent reporting of procedures and data would aide in making useful comparisons between studies.

#### 3.2.1 Auditory processing-MEG

The comparison of auditory research contains similar issues to those within the visual literature, with stimuli ranging from simple sound presentation (Lengle et al., 2001) to an assessment of auditory discrimination ability interpreted as indicating higher-level cognitive abilities of the foetus. Researchers have variously interpreted their findings as evidence for short-term memory capacities (Huotilainen et al., 2005), language learning (Draganova et al., 2007) and speech perception and development (Draganova et al., 2005).

In our comparisons of methodologies investigating foetal response to auditory stimuli using fMEG, it is clear that the chosen frequency of tones is relatively consistent across studies. Generally levels of 500, 750, 1000 Hz are presented to the foetus. Decibel level is also relatively consistent across studies. Studies all used between 100 dB and 120 dB with one using 90 dB (Huotilainen et al., 2005). This is also the case for fMRI research. This is likely due to safety level recommendations of foetal exposure to auditory stimuli. Graven (2000) recommends avoidance of exposure of the foetus to sound levels below that of 250 Hz, above 65 dB and reports physiological motor response to sound from 70 dB. Maternal tissue has little attenuation impact on sounds below that of 250 Hz but is estimated to attenuate sounds between 0.5 and 2 kHz by 20 dB (Gerhardt and Abrams, 2000). This leads to a relatively small window of flexibility in frequency and dB level as the two must be balanced in order to present an audible, yet safe level of sound.

Within the field there is substantial variation in the number of presentations or duration of auditory stimuli. Studies have presented as few as 100 stimuli, up to over 700 stimuli ranging from 20 ms to 1000 ms in duration. There is some variation in total duration of study, however, since there is no apparent pattern between those reporting different levels of response rate, it is concluded that this is not likely have an effect on auditory response rate.

The delivery method of the auditory stimulus is likely to have an impact on the resulting response rate. A number of studies (Lengle et al., 2001, Zappasodi et al., 2001, Schneider et al., 2001) classified within the “moderate response rate” category placed a speaker outside the magnetically shielded room and delivered sound to the abdomen via a tube filtered through to the testing room. Others (Preissl et al., 2004, Draganova et al., 2005, Eswaran et al., 2005, Draganova et al., 2007) in this category used a tube in combination with an air filled bag placed at the abdomen to deliver the auditory stimuli. In contrast, 4 out of 5 of those in the “good” category used the tube and air-filled bag combination. This difference between studies points toward potential effects of stimulus delivery in terms of the quality of obtained data.

The inter-stimulus interval is unlikely to have an effect on the resulting response rate. This is found both within studies, with no difference on detection rate for varying intervals (Preissl et al., 2004, Draganova et al., 2005, Draganova et al., 2007), and in the comparisons made here between studies. The relationship between inter-stimulus interval and stimulus duration, could, however, have a significant impact on the response rate of the foetus and has been calculated for all studies. Those in the “good” response rate category had a ratio of 1:2–1:4 stimulus duration to interval. Those with a “moderate” response rate had much higher ratios of between 1:8 and 1:25. One interpretation of this is that the foetus is more stimulated by a larger ratio of stimuli to non-stimuli periods. This could avoid the foetus moving into a deeper sleep state and therefore will allow for a better response rate across the study. Draganova et al. (2005) varied this ratio between 1:5 and 1:11 since inter-stimulus interval varied between 800 ms and 1200 ms. Though no significant differences were found between intervals, the relationship could be further explored with varying inter-stimulus intervals as well as varying stimulus duration in order to establish the effect of the relationship between the two on foetal response rate.

A number of studies have reported a significant decrease in latency in response to auditory stimuli with gestational age (Lengle et al., 2001, Schneider et al., 2001, Schleussner et al., 2004, Holst et al., 2005, Schleger et al., 2014). In contrast, Sheridan et al., 2010 only reported a trend for this result while Draganova et al. (2007) did not find a significant difference in latency with gestational age. The latter studies used an oddball paradigm while, with the exception of Holst et al. (2005), all papers reporting a significant age effect used a simple pure tone paradigm. It is possible that the more complex oddball paradigm masked any age effects of latency to respond to the auditory stimuli. Alternatively, this difference in reporting of an age effect could be the result of the two different kinds of paradigms and foetal response rate may be negatively correlated with age in response to simple paradigms only. While Draganova et al. (2007) showed a poor response rate by our calculations, the number of accepted participants is comparable to studies that successfully show a significant age effect. Additionally, this paper reported that 600 standard trials and 70 deviant trials were analysed, though this cannot be compared with the other two papers as they did not report the number of trials included in the analysis post artefact removal. Therefore, those who reported a failure to find a significant age effect did not appear to do so due to a lack of statistical power. However, more consistent reporting of the number of trials included in the analysis would facilitate the quality of the field.

Additionally, there is evidence of the failure to investigate any age effects despite an indication that this is an achievable analysis. For example, Eswaran et al. (2005) had 11 participants from 28 weeks GA onwards and 21 included recordings. There is therefore the potential for this study to have had a large enough age range and enough power for an age effect calculation. It must be considered that, as negative results are rarely reported, the age effect could be a less consistent finding than the literature currently portrays. Publication bias could be having an effect on comparisons that can be made on this topic (Sterne & Egger, 2001).

Few auditory studies focus on a specific age range. Many feature a large age range from around 28–40 weeks GA without differentiating GA in their analysis. This could be the result of power issues due to small sample sizes but some report a general latency of 100–300 ms latency with a significant (or trend towards) reduction in latency with age. Though these studies do not make predictions of response latency for any particular GA, they do support the observations made between studies of smaller age ranges. A detailed analysis of functional capacity at specific gestational ages requires more focussed age ranges than those present in the current literature.

When examining the variation between studies in the latency of response to auditory stimuli, studies with an age range sample of 30–35 GA tended to report a latency of 200–300 ms. Studies with a sample of around 35–40 GA tended to report a faster latency of 100–200 ms. It is possible that this is a developmental effect that illustrates improved efficiency of the processing of stimuli over late gestation. Another, not necessarily opposing, interpretation is that during late gestation, there is an emergence of an additional dominant component for auditory processing. Schleussner and Schneider (2004) found a p2pm component at a latency of 144–199 ms, which could correspond to the latencies found in later gestation. This highlights an advantage to the use of either (a) a large sample in a longitudinal design or (b) a small age range as questions can now be asked as to the developmental trajectory of auditory processing in the foetus.

Similarly to Draganova et al. (2005) who reported on a younger GA 33–36 weeks, other studies report either a single or additional late discriminatory response (LDN) with a latency of at least 400 ms. Recently, Schleger et al. (2014) published a study in which they found foetal response latency of 427 ms. Due to the subject in question, numeracy, a unique design was used insofar as two tones were compared with four tones. This allowed for a discrimination detection response to either the third or the fourth tone thereby allowing more time for a late response. Other studies have provided evidence of the LDN response with the more typically used standard intermixed with deviant tone oddball presentation of stimuli (Schleussner et al., 2001, Draganova et al., 2005). Therefore, it seems this late response can be seen across discriminatory presentation procedures.

### 3.3 Auditory processing–fMRI

For fMRI, recorded response rates were between 50 and 70%, which is similar to fMEG studies. However, similar to visual studies, fMRI research featured smaller sample sizes (3–15) and more restricted age ranges (1–4 weeks) when contrasted with fMEG. More sophisticated auditory stimuli have also been used, ranging from Spanish guitar music (Moore et al., 2001) to the discrimination of familiar and unfamiliar speech (Jardri et al., 2012). There was a consistent report of unilateral, left temporal activation bias (Jardri et al., 2008, Jardri et al., 2012). However, one study reported mixed results with 3 participants displaying a left bias and 2 exhibiting a right (Moore et al., 2001). This study was the only study to use music as stimulus compared with simple tones, speech or vibroacoustic stimuli. It might be the case that at this stage of development, music is processed differently to other auditory stimuli. Certainly, differential processing has been found for maternal speech when contrasted with speech of an unfamiliar female voice, resulting in different areas of activation within the temporal cortex (Jardri et al., 2012). Moore et al. (2001) also featured stimuli presented at 85 dB, the lowest reported. One possibility is that this quieter presentation can cause more variation in processing, when contrasted with other studies. Two further studies failed to shed light on the consistency of unilateral activation in the left hemisphere, with (Hykin et al., 1999) reporting a unilateral response yet not stating the actual hemisphere, and (Fulford et al., 2004) reporting activation of the temporal cortex but not providing specific information. Studies using fMRI found a similar response rate, overall, to those utilising MEG using more complex auditory stimuli.

Given that both fMRI and fMEG studies follow safety guidelines, the auditory stimuli that have been presented are consistent within and between each other in the use of 0.5–1 KHz and 90–120 dB. Similar response rates are reported for the two techniques. Specific developmental progression of auditory processing has been better tracked with fMRI through the use of smaller GA ranges, although larger sample sizes would improve the quality of obtained data. The increase in sophistication of equipment and stimulus delivery methods has predictably allowed better response rates in fMEG studies. Additionally, stimulus to ISI ratio, number of presentations and total length of study have been discussed as potential causes for variations in response rates and latency. We suggest that these parameters could be standardised between researchers across studies in order to permit a comparison of data collected from various samples and labs. Furthermore, we argue that more description is required in terms of parameters and results in order to assist comparisons of published results, such as response range and response average. We propose that larger sample sizes and the comparison between smaller age ranges could help to determine developmental trajectories related with foetal auditory development.

## 4 Discussion

Variation in the reports of foetal neural response rate and latency may well be explained through patterns across the designs of studies in the field. Variations in brightness of light stimuli, area of illumination, number of presentations and length of stimuli alongside ISI to stimulus duration have been discussed in terms of their potential effects on cortical response. The developmental trajectory of response remains particularly unclear given the lack of categorisation of ages in studies using a large GA range. Missing data due to under reporting of analysis techniques and specific aspects of procedures makes comparisons within this field difficult. Clear reporting of the percentage or number of participants or number of clean trials remaining post artefact removal alongside the percentage of response from remaining data is very rarely reported with Zappasodi et al. (2001) being one of the few studies to present these factors and Schleussner et al. (2004), for example, failing to report any. On the other hand, while Draganova et al. (2005) did not discuss percentage of clean data, this paper did discuss response rates for two levels of criteria; simple response and discriminatory response. This is useful information that is only found in this study. The total length of the study is also omitted in most studies (e.g., Schleussner et al., 2004, Huotilainen et al., 2005) making it difficult to assess attention as a cause of response rate variation. There is, however, some level of consistency between fMRI and fMEG studies. Volume and pitch levels presented are generally similar across auditory studies and stimulus duration is generally consistent across visual studies. Furthermore, response rates generally do not differ between fMRI and fMEG, suggesting equivalent sensitivity to functional response in different samples of foetuses. Regarding the reporting of methodology in studies, given the variability of which factors are reported, we argue that to facilitate comparisons of data, more aspects of stimulus presentation should be detailed. The investigation of each aspect in its effect of cortical response should be included in future publications in the field. Additionally, given the importance of artefacts, future research needs to control for foetal eye movements when analysing foetal responses to visual stimuli. Recent high definition 4D ultrasound technology is likely to lessen some of the difficulties researchers have previously faced in assessing this factor. It should be acknowledged that the investigation of the functional foetal brain is currently at an early stage and is only beginning to move beyond the first attempts that were made by Blum et al. (1985). This area of research has been largely constrained by cost and availability of equipment. Current technological advances provide opportunities for more research in this area of early development.

## Conflict of interest

The authors declare that there is no conflict of interest.
